# Predicting morphological and functional variations of benign adrenal incidentalomas in relation to initial characteristics

**DOI:** 10.3389/fendo.2023.1179817

**Published:** 2023-06-06

**Authors:** Chiara Parazzoli, Vittoria Favero, Carmen Aresta, Valentina Morelli

**Affiliations:** ^1^ Department of Medical Biotechnology and Translational Medicine, University of Milan, Milan, Italy; ^2^ Endocrinology Department & Lab of Endocrine and Metabolic Research, IRCCS-Istituto Auxologico Italiano, Milan, Italy

**Keywords:** adrenal incidentaloma, autonomous cortisol secretion, non-functional adrenal incidentaloma, attenuation value, Cushing’s syndrome

## Abstract

The follow-up strategy for unresected non-functional adrenal tumors (NFAT) is a major controversial issue in endocrinological clinical practice, as the natural history of adrenal incidentalomas (AI) is partially unknown and a consensus on their adequate management is lacking. In a recent longitudinal study by Ceccato et al., a large cohort of patients with conservatively treated AI were evaluated for possible radiological variations over time and their relationship with autonomous cortisol secretion (ACS). Starting from this paper, we performed a literature review of available longitudinal studies focus on the same issue. Notwithstanding the high variability in the duration of follow-up and in the criteria used to define ACS in the included studies, our findings support the idea that there is a not negligible risk of morphological and functional changes, which may have metabolic implications, especially after 5-10 years of follow-up. Unfortunately, these variations seem to be scarcely predictable. Therefore, it may be risky to interrupt the follow-up in patients with NFAT, in particular in the presence of larger diameter of the adenoma and higher cortisol levels at diagnosis. These results should be considered in defining the optimal management of these patients.

## Introduction

Adrenal incidentalomas (AI) are asymptomatic adrenal masses, greater than 1 cm in diameter, detected on imaging studies performed for other reasons than adrenal disease (1). Their incidence has increased particularly in the last two decades, most likely due to the spread and improvement of modern abdominal computed tomography (CT) and magnetic resonance imaging scanning technology in clinical practice (2). Indeed, the most recent AI series reported a prevalence of around 5% to 7% in radiological studies (3, 4), estimated to be even higher in adults over 65 years of age (2). According to the current guidelines of European Society of Endocrinology, at the time of initial diagnosis all patients with AI should undergo a careful clinical, endocrine biochemical and imaging evaluation to assess whether the tumor is malignant and to determine the presence of excess adrenal hormones (1). Several recommendations for the management of AI have been proposed. For adrenal masses <4 cm with clear benign features, defined by homogeneous consistency and lipid-rich with a density ≤10 Hounsfield Units (HU) on an unenhanced CT scan, and normal hormones activity at initial work-up no further investigations are necessary due to the low risk of malignant and/or functional transformation (1). Indeed, the most common AI are non-functioning adrenocortical tumors (NFAT), with a prevalence of 70-85% (2, 5, 6). These types of lesions are usually asymptomatic and radiologically benign, and the majority of cases are managed conservatively. One of the most controversial issues in this context is the follow-up strategy for unresected NFAT. A recent systematic review and meta-analysis of approximately 3000 patients with NFAT confirmed that after a mean follow-up of over 3 years, no cases developed adrenal cancer and only 4.3% of patients showed growth in size and hormonal activity (7). This supports the view that this type of lesion does not require follow-up. In contrast, other guidelines (8) or consensus positions (9–11) recommend maintaining radiological and biochemical follow-up of NFAT, regardless of their characteristics, due to the possibility of changes in their nature when are followed for a long period. In fact, relatively few studies really addressed long-term follow-up of unresected AI, making the usefulness and possible timing of reassessment over time unclear. Moreover, this issue is becoming increasingly important in clinical practice, given the increasing prevalence of NFAT and the need to contain health care costs. In order to manage NFAT more effectively, it would be useful to identify at initial diagnosis which lesions will tend to evolve over time in terms of radiological features and/or hormonal activity and which will remain stable. In this respect, a recent study by Ceccato et al. evaluated a large cohort of conservatively managed AI to detect variations in size and attenuation value over time and their possible relationship with autonomic cortisol secretion (ACS). Based on their results, the authors suggest that follow-up imaging should be performed 5 years after diagnosis, especially in patients with ACS, lipid-poor adenomas and a large diameter at baseline (12). In this mini-review, we evaluated other studies available in the literature to find out if any clinical or biochemical characteristic may be associated with radiological changes and the development of ACS in benign adrenal adenomas over time. This would allow to schedule a tailored follow-up of patients with unresected NFAT, with a favorable cost-benefit ratio.

## Methods

We searched the PubMed and MEDLINE databases to identify articles describing radiological and/or functional follow-up of AI. We used the following keywords and/or their combinations: AI, non-functional, autonomous cortisol secretion, Cushing’s syndrome, follow-up, longitudinal, attenuation value, adenoma size, mass enlargement, endocrine hyperfunction, predictive factors. Original articles evaluating predictive criteria for morphological and/or functional modification in NFAT with at least 2 years of follow-up were included. We initially identified 20 studies with adequate observation period. Of these, only 14 were included in our analysis because they reported the predictive criteria this review was focused on. The list of included studies is shown in **Table 1**.

**Table 1:** Longitudinal studies with at least 2 years of FU evaluating predictive parameters of adrenal mass enlargement of cortisol secretion progression.

Author, year	Predictive criteria at diagnosis of function variation	Predictive criteria at diagnosis of diameter increase
Barzon L et al, 1999 (13)	- adenoma size >3 cm - exclusive or asymmetric scintigraphic uptake - older age (≥56 yrs) - female gender - hypertension	- abnormal endocrine tests - older age (≥56 yrs)
Comlekci A et al, 2001 (14)	- age >40 yrs	- age >40 yrs
Libè R et al, 2002 (15)	*Not associated with:* - adenoma size	- normal adrenal function
Bernini GP et al, 2005 (16)	*Not associated with:* - adenoma side - adenoma size - gender - BMI - T2DM - hypertension	*Not associated with* - adenoma side - adenoma size - gender - BMI - endocrine pattern - T2DM - hypertension
Fagour A et al, 2009 (17)	- unilateral radiocholesterol uptake - post-1mgDST cortisol level >50 nmol/L *Not associated with:* - adenoma size - obesity - T2DM - hypertension - midnight cortisol ≥116 nmol/L - ACTH <2.2 pmol/L	*Not associated with:* - radiocholesterol uptake
Cho YY et al, 2013 (18)	Not evaluated	*Not associated with:* - age - gender - BMI - adenoma size - HU value
Hong AR et al, 2017 (19)	- older age *Not associated with:* - gender - adenoma size - T2DM - hypertension - cardiovascular disease	*Not associated with:* - adenoma size
Papanastasiou L et al, 2017 (20)	- higher post-LDDST serum cortisol levels	- ACS
Kim J et al, 2020 (21)	Not evaluated	- T2DM
Yilmaz N et al, 2020 (20)	*Not associated with:* - adenoma size	- CS - radiological diagnosis of myelolipoma
Podbregar A et al, 2021 (23)	- post-1mgDST cortisol levels >30 nmol/L - BMI >25 kg/m^2^ - higher heart rate - higher fasting glucose	*Not associated with:* - adenoma size - BMI
Araujo−Castro M et al, 2021 (24)	*Not associated with:* - adenoma size - bilateral adenomas	*Not associated with:* - adenoma size - bilateral adenomas - post-1mgDST serum cortisol levels
Falcetta P et al, 2021 (25)	- adenoma size ≥2.8 cm - bilateral adenomas - ACTH <2.2 pmol/L - IFG - stroke - atherosclerosis	- lower BMI - higher post-1mg DST serum cortisol levels
Ceccato F et al, 2021 (12)	- adenoma size >2.4 cm	- adenoma size >2.4 cm - HU >10

FU, follow-up; yrs, years; BMI, body mass index; T2DM, type 2 diabetes mellitus; 1mgDST, 1-mg overnight dexamethasone suppression test; ACTH, adrenocorticotropic hormone levels; HU, Hounsfield Unit; LDDS, low-dose dexamethasone suppression test; ACS, autonomous cortisol secretion; CS, Cushing’s Syndrome; IFG, impaired fasting glucose.

### Predictive criteria of diameter increase

The main point addressed by the paper of Ceccato et al. concerns the radiological modifications (diameter and lipid content) in a large cohort of patients with AI, according to their cortisol secretion, after a long-term follow-up (12). So far, other longitudinal studies have addressed the same topic. Principal characteristics of the included articles are summarized in **Supplementary Table 1**. However, Ceccato’s study is the first to investigate changes in attenuation values of AI in relation to morphological and functional variations over time.

Firstly, in their paper the authors found that, after a median observation period of 52 months, the increase in diameter of apparently benign AI was minimal (Δ+1 mm from baseline) (12). A greater enlargement >1 cm was found in 3.3% of cases, but no malignancies have been reported. Considering other previous studies, a growth rate of at least 1 cm was reported in 2-15% of cases always without any malignant transformation (13, 14, 20, 22–24). Overall, a minimal increase in diameter was found in a very variable percentage of cases, ranging from 10 to 56.7% (13–18, 20, 22, 23, 25).

**Figure 1:**
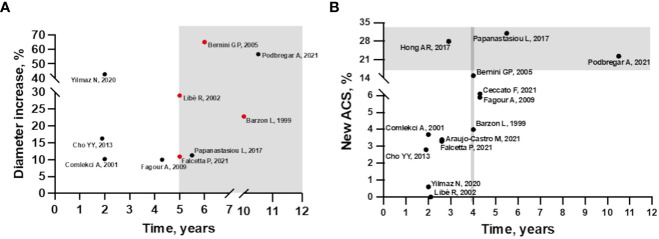
**(A)** Incidence of mass enlargement during follow-up in patients with non-functioning adrenal incidentaloma. The increase in adenoma size occurs mainly after 5 years of follow-up (gray area). Cumulative risk of mass increase in red. **(B)** Incidence of autonomous cortisol secretion during follow-up in patients with non-functioning adrenal incidentaloma at baseline. In studies with 4 years of follow-up, the incidence of ACS starts to increase (area delimited by grey line), but most cases occur after 5 years of follow-up (grey area), except for the study by Hong et al.

In fact, the longer is the follow-up, the greater is the growth of the AI, as observed by Ceccato et al. (12). In particular, in their study adenoma growth was significant, albeit modest, when the follow-up CT scan was performed at least 5 years from diagnosis. Other authors have also found that the likelihood of mass enlargement increases with the duration of follow-up (13, 15, 19, 22, 25) (**Figure 1A**). Specifically, in the study by Barzon et al. the cumulative risk of mass enlargement increased from 8% after 1 year to 22.8% after 10 years (13). Similarly, Libè et al. and Falcetta et al. reported that this risk was 29% and 10.9%, respectively, after 5 years (15, 25), while Bernini et al. estimated that the cumulative risk of mass enlargement was globally elevated, around 65%, and progressive up to 80 months (16). In contrast, another recent large study found that the increase in adenoma size was already greater after 2 years of follow-up (22).

Regarding baseline predictive criteria of adenomas size variations, Ceccato et al. found that an initial diameter >2.4 cm was associated to a larger increase over time (12). So far, none of the other studies investigating this issue have found this association before (16, 18, 19, 23, 24).

Furthermore, some authors have evaluated a possible relationship between hormonal activity at baseline and mass enlargement over time, but the results have been contradictory. Indeed, several studies have correlated the enlargement of the mass with the presence of initial abnormal endocrine tests (13, 20, 22, 25) whereas in one study the increase in adenoma diameter was predicted by normal adrenal function at baseline (15). Finally, in the study by Ceccato et al. and others, the increase in diameter was independent of hormone secretion (12, 16, 24).

Among the other predictive criteria summarized in **Table 1**, it was observed that older age (specifically ages >40 and 56 years) was associated with an increase in adenoma diameter over time (13, 14). In addition, an association between certain cardiovascular risk factors and/or metabolic complications at baseline with tumor growth was investigated (16, 18, 21, 23, 25). Interestingly, only Kim et al. found an association with type 2 diabetes mellitus (T2DM) (21) and Falcetta et al. with lower BMI (25).

Finally, the novelty of Ceccato’ study is the evaluation of attenuation values as a predictive criterion for tumor growth. In fact, despite almost all included studies stated that they considered only benign AI, mean HU values were rarely reported (18). In their study, mean density at baseline was higher in patients with ACS than in those with NFAT. A decrease in the attenuation value (-4 HU) was detected in 101 cases at least after 60 months. A negative regression between the duration of follow-up and attenuation value was observed, especially in patients with ACS: up to 70% of them had a lower mean density than at baseline. Moreover, the authors found for the first time that AI with HU >10 at baseline presented an increase in size and the greatest decrease in attenuation value during follow-up (12).

### Predictive criteria of functional variation

Although the paper by Ceccato et al. focuses primarily on radiological modifications in patients with AI, the authors also evaluated variations in cortisol secretion over time. In particular, in their cohort, none of the 181 AI patients studied progressed to over Cushing’s syndrome (CS), whereas 11 patients (6.2%) developed ACS after approximately 4 years (12). As ACS is a condition characterized by the absence of the classic features of CS, in the absence of follow-up these 11 patients would have been lost, with important implications. In fact, in contrast to NFAT, AI patients with ACS should be followed up over time (1) because this condition, despite having milder cortisol levels than CS, can still lead to several comorbidities of overt hypercortisolism with increased cardiovascular and mortality risk (26).

Overall, considering other available longitudinal studies, the incidence of new cases of ACS from NFAT at diagnosis is relatively low but characterized by high variability (0-31%) (12–25) (**Figure 1B**). This variability may be explained by the lack of consensus on the diagnostic definition of ACS. In most of the analyzed studies, ACS was defined by incomplete cortisol suppression in response to the 1 mg dexamethasone overnight suppression test (1mgDST), in the absence of specific clinical signs of CS. The rate of new cases was higher when the 50 nmol/L threshold was used (12, 16, 18–20, 23) than in studies using 138 nmol/L (22, 24, 25) or 1mgDST associated with abnormalities in other elements of the hypothalamic-pituitary-adrenal (HPA) axis, such as low adrenocorticotropic hormone (ACTH) and dehydroepiandrosterone sulphate (DHEAS) levels, high urinary free cortisol (UFC) and high midnight cortisol levels (13–15, 17, 22). Details of incidence rates and criteria for defining ACS and CS are described in **Supplementary Table 1.**

Moreover, collected data support the evidence that the development of CS over time is very rare and more common in patients with ACS at baseline (0-2.6%) (12–16, 18–25). Only one study showed an incidence of 6% but had defined CS solely by the presence of classic clinical signs (17). Regarding other clinically relevant hormonal excesses, although not systematically studied, the development of phaeochromocytoma was reported in 4 of the studies evaluated (13, 18, 25, 27) and one case of primary aldosteronism was identified (25).

Several NFAT elements at baseline were found to be associated with the occurrence of hypercortisolism over time, helping to identify which masses need to be monitored more carefully (**Table 1** **)**.

Ceccato et al. observed that an initial diameter >2.4 cm in NFAT was able to predict not only mass enlargement, but also the development of ACS over time (12). In agreement with them, Barzon et al. (13) and Falcetta et al. (25) reported that adenoma mass sizes >3 cm and ≥2.8 cm were predictive of ACS, respectively. However, the same association was not confirmed by other authors (15–17, 19, 22, 24). Another radiological feature frequently studied was the site of the lesion (16, 24, 25) and conflicting results emerged as only Falcetta et al. found that bilateral AI was associated with the occurrence of ACS over time (25).

On the other hand, more consensus has been found in the literature to consider hormonal parameters of the HPA axis as predictors of functional changes. In particular, for many authors, higher cortisol levels but still within the normal range, after the 1mgDST (17, 23) or the low-dose dexamethasone suppression test (LDDST) (20) at baseline were able to predict the development of ACS. Specifically, Podbregar studied a cohort of 67 NFAT for 10 years and identified that an initial cortisol level post 1mgDST of 30 nmol/L was the threshold to distinguish NFAT who developed ACS from those who remained non-functional (23). Instead, according to Falcetta et al. (25) but in disagreement with a previous smaller study (17), this risk was associated with the presence of low ACTH (<2.2 pmol/L) at the time of detection.

It is also interesting to note that a higher prevalence of various cardiovascular risk factors and metabolic complications at baseline was detected in patients who developed hormonal activity during follow-up than in those who remained non-functional. In particular, hypertension (13) and higher heart rate (23), impaired fasting glucose (IFG) (25) or higher fasting glucose levels, overweight and obesity (23), stroke and atherosclerosis (25) were found to be predictive factors for the occurrence of ACS. However, other evidence disagreed with them (16, 17, 19).

A further consideration concerns older age as a predictor of functional change. In fact, in the study of Barzon et al. and Comlekci et al. an age >40 and 56 years, respectively, was associated with higher risk not only of increasing adenoma size but also of developing ACS over time (13, 14), and it was confirmed in more recent findings (19).

Finally, Ceccato et al. highlight the importance of the follow-up duration confirming that long-term follow-up is related to the risk of developing ACS as well as to tumor growth (12). In agreement with them, Falcetta et al. and Libè et al. confirmed that the cumulative risk of developing an ACS increases with time and is higher after 5 years (15, 25).

## Discussion

The detection of AI has become increasingly common in recent years due to the widespread use of imaging studies. The majority of these lesions are NFAT, for which conservative management is suggested. Current guidelines do not recommend systematic radiological and biochemical follow-up in this type of lesion due to the low risk of malignant transformation and development of hormonal hypersecretion (1). However, the follow-up strategy for unresected NFAT remains one of the most controversial issues, as natural history of adrenal lesions is still partially unknown. Based on the longitudinal study by Ceccato et al. (12) we reviewed papers with at least 2 years of follow-up analyzing the predictive factors of morphological and/or functional modifications of NFAT over time.

Firstly, this literature review remarks that AI with initially benign radiological features do not grow significantly over time (increase diameter >1cm from 2% to 15% (12–14, 20, 22–24)) and the progression to malignancy does not appear to be a concern. Regarding hormone activity, the incidence of new cases of ACS is relatively low in the majority of studies, even if in one of them it occurred in up to 31% of patients (20). On the contrary, the progression to CS has been confirmed to be rare. Concerning the possible secretion of other adrenal hormones, only a few studies have included this data. Nevertheless, it is worth mentioning that 4 different studies (13, 18, 25, 27) reported during the follow-up the development of pheochromocytoma from NFAT, and one of them also found a case of primary aldosteronism (25). It is possible that pheochromocytoma was already present at the time of diagnosis but unrecognized.

Regarding predictive criteria, a correlation with the duration of follow-up was found for both radiological and functional modification. Several studies have suggested that the risk of mass enlargement and of developing ACS increases with longer follow-up, especially after 5-10 years of follow-up (**Figures 1A, B**). However, it is important to note that data available after 10 years of observation are very scarce. So far, only one study has a mean follow-up period of 10.5 years, and it was the one with the largest number of patients who experienced an increase in diameter. Unfortunately, this study included a limited number of patients (23). At the other side, the largest series [more than 300 patients (19, 22, 25) even a cohort of 621 (24)] had a median follow-up of <3 years, limiting the usefulness of these data. The strength of the study by Ceccato et al. in here discussed, is that it included 181 patients with a median follow-up period of 52 months. Moreover, the radiological evaluations performed were more accurate than in other studies. This may explain why, differently from other studies, a clear correlation between initial adenoma diameter and morphological and functional progression was found (12). Despite the importance of attenuation values in defining the nature of AI is well known, this is the first study exploring a correlation between radiological characteristics and progression of NFAT, confirming that lesions with HU >10 should be monitored more carefully.

Interestingly, morphological and functional changes over time seem to be associated one to each other. Specifically, the study by Papanastasiou et al. demonstrated that the adenoma size increase was correlated with the development of ACS, thus suggesting the need of biochemical re-evaluation in case of mass enlargement (20). However, this association has not been confirmed by other authors (22, 28).

In this regard, it is possible that in the next future, a better initial biochemical characterization of NFAT by the use of mass spectrometric–based measurements of panels of steroids, could be useful in identifying patients at higher risk of disease progression (29). So far, no data are available about the use of these techniques, which are not widely available, in this setting.

Currently, there is poor consensus among the different studies on predictive criteria identified. However, it seems that adrenal adenomas larger than 2.4 cm could deserve more attention, in particular for the risk of functional progression. In fact, ACS may occur in a not negligible percentages of patients, mainly in the presence of a larger initial adenoma diameter (12, 13, 25), higher cortisol levels after 1mDST or LDDST at baseline (17, 20, 23), and cardiovascular risk factors (13, 23, 25). This last aspect is important because cardiovascular and metabolic comorbidities may be exacerbated by the occurrence of cortisol hypersecretion.

Furthermore, it was found that older age can predict both morphological and functional changes (13, 14), however, it is not surprising given the mean age of patients with AI. It could also be hypothesized that younger patients with larger nodules were more frequently addressed to surgery and consequently not included in longitudinal studies. Moreover, it is important to notice that a potential bias affecting all the evaluated studies is the patients drop-out during follow-up. Reasons for this inconvenient are various and not always specified. However, it can be assumed that some patients who underwent adrenalectomy because of increased mass size and/or development of hormonal activity, were not encountered in the final evaluation thus affecting results.

In conclusion, notwithstanding all these limitations, our literature review suggests that the risk of morphological and functional changes of AI increases over time, especially after 5-10 years of follow-up, even if currently it is scarcely predictable. Therefore, to interrupt the follow-up may be risky in patients with NFAT. All these aspects should be considered to define the optimal management of these patients.

## Author contributions

All authors did data collection, writing, and critical revision of the article. All authors contributed to the article and approved the submitted version.
