# Supplementary material for: Predicting morphological and functional variations of benign adrenal incidentalomas in relation to initial characteristics
Source: Front Endocrinol (Lausanne). 2023 Jun 6;14:1179817. doi: 10.3389/fendo.2023.1179817 (PMC10280065; doi:10.3389/fendo.2023.1179817)
Supplement: Supplementary file 1 [file Table_1.docx]

**Table 1. Longitudinal studies with at least 2 years of FU**

| **Author,**  **year** | **Patients, n**  **(f/m)** | **ACS, n (%)** | **Criteria of ACS** | **Diameter cm, mean±SD (range) or median (IQR)** | **FU, mean±SD (range) or median (IQR)** | **New cases of ACS, n (%)**  **new cases of CS, n (%)** | **Diameter increase, n (%)** | **ΔDiameter** |
| --- | --- | --- | --- | --- | --- | --- | --- | --- |
| **Barzon L et al, 1999** | 75 (52/23) | 0 | Absence of clinical features of CS, 1mgDST >140 nmol/L, and at least one of the following:  (i) impaired cortisol rhythm  (ii) ↑UFC  (iii) ACTH <4 pmol/L | 2.5±1.0  (1.0-5.6) | Median 4 (2-10) yrs | 3/75 (4%)  2/75 (2.6%) | 11/75 (15%) | ≥1 cm |
| **Comlekci A et al, 2001** | 376 (266/110)  Follow-up:  - biochemical 162 patients  - morphological 293 patients | 41 (10.9%) | Absence of clinical features of CS, cortisol level post 1mg-DST >50 nmol/L and at least one of the following:  (i) ACTH <1.1 pmol/L  (ii) ↑UFC  (iii) midnight cortisol >7.5 µg/dL | 2.5 (0.7-9.7) | Median 24 mth | 6/162 (3.7%)  0/162 (0%) | 30/293 (10.2%) | <1 cm in 20 patients  ≥1cm in 10 patients |
| **Libè R et al, 2002** | 64 (40/24) | 12 (18.7%) | Absence of clinical features of CS and at least two of the following:  (i) cortisol level post 1mgDST >138 nmol/L  (ii) UFC ≥275 nmol/24h  (iii) ACTH <0.7 pmol/L  (iv) ACTH after CRH test <4.4 pmol/L  (v) cortisol level after opioid agonist loperamide administration | 2.5±0.1  (1.0-4.0) | Median 25.5 (12-120) mth | 0/64 (0%)  0/64 (0%) | 13/64 (20%) | 1.7±0.2 cm |
| **Bernini GP et al, 2005** | 115 (72/43) | 23 (20%)  11 cases with cortisol level post 1mgDST >50 nmol/L | Abnormality concerned the HPA axis: ACTH <2 pmol/L, ↓DHEAS, ↑cortisol level, cortisol level post 1mgDST >50 nmol/L or combinations of these | 2.5±0.09 | Median 4 (1-7) yrs | 17*/115 (15%)  3 cases with cortisol level post 1mgDST >50 nmol/L  0/115 (0%) | 32/115 (27.8%) | <0.5 cm in 2/3 of patients |
| **Fagour A et al, 2009** | 51 (28/23) | 24 (47%) | Cortisol level post 1mgDST >50 nmol/l and at least two of the following:  (i) ACTH ≤2 pmol/L  (ii) cortisol percent ratio ≥50%  (iii) elevated midnight plasma cortisol ≥116 nmol/L | 2.4±0.8 | Mean 4.3±1.6 yrs | 3/51 (5.9%)  3/51 (6%) | 5/51 (10%) | 1 cm |
| **Cho YY et al, 2013** | 282 (110/172)  Follow-up:  - biochemical 72 patients  - morphological 147 patients | 28 (9.9%) | Cortisol level post 1mgDST >55 nmol/L | 2.3±1.4 | Mean 23.1 mth | 2/72 (2.8%)  0/72 (0%) | 24/147 (16.3%) | ≤1 cm |
| **Hong AR et al, 2017** | 1149 (518/631)  Follow-up:  - biochemical 193 patients  - morphological 449 patients | 82 (7.1%) | Absence of clinical features of CS and cortisol level post 1mgDST ≥50 nmol/L | 1.8 (1.3-2.7) | Biochemical: mean 2.9 (1.6-4.9) yrs  Morphological: mean 4.31±2.76 yrs | 54/193 (28%)  0/193 (0%) | n.a. | 0.14±0.34 |
| **Papanastasiou L et al, 2017** | 71 (48/23)  Follow-up:  - biochemical 51 patients | 20 (28%) | Cortisol level post LDDST ≥50 nmol/L | 2.1±0.8 | Mean 5.54±1.7 yrs | 16/51 (31%)  0/51 (0%) | 8/71 (11.3%) | >0.5 cm in 7 patients  >1.5 cm in 1 patient |
| **Kim J et al, 2020** | 154 (40/114) | 0 | Cortisol level post 1mgDST ≥50 nmol/L | 1.6±0.7 | Mean 6.0±3.5 yrs | Not evaluated | n.a. | 0.2±0.4 cm |
| **Yilmaz N et al, 2020** | 755 (497/258)  Follow-up:  biochemical and  morphological 325 patients | 37 (4.9%) | Cortisol level post LDDST ≥138 nmol/L | 2.1 (1.0-1.9) | Median 24 (6-120) mth | 2/325 (0.6 %)  2/325 (0.6 %) | 139/325 (42.8%) | <1 cm in 101 patients  ≥1 cm in 38 patients |
| **Podbregar A et al, 2021** | 67 (47/20) | 0 | Cortisol level post 1mgDST ≥50 nmol/L | Right-sided: 2.5 (1.5-2.8)  Left-sided  2.0 (1.4-2.8) | Mean 10.5 (9.1-11.9) yrs | 15/67 (22.4%)  0/67 (0%) | 38/67 (56.7%) | <1 cm  ≥1 cm in 6 patients |
| **Araujo‑Castro M et al, 2021** | 823 (472/ 351)  Follow-up:  - biochemical 437 patients  - morphological 621 patients | 47 (5.7%) | Absence of clinical features of CS and cortisol level post 1mgDST ≥138 nmol/L | 2.1±1.1 | Median 31.2 (14.4-56.5) mth | 15/437 (3.4%)  0/437 (0%) | n.a. | Unilateral AI 0.0±0.03 cm  Bilateral AI 0.1±0.06 cm  >1 cm in 13 patients |
| **Falcetta P et al, 2021** | 310 (200/110)  Follow-up:  - biochemical 209 patients | 81 (26.1%) | Absence of clinical features of CS and cortisol level post 1 mgDST >138 nmol/L or cortisol level post 1mgDST between 50-138 nmol/L and at least one of the following:  (i) ACTH <2.2 pmol/L  (ii) UFC ≥1117.8 mmol/24h  (iii) midnight cortisol >220 nmol/L  (iv) cortisol level post-LDDST >50 nmol/L | 2.2±1.0 | Median 31.4 (13.0-78.6) mth | 7/209 (3.3%)  2/209 (1%) | 53/310 (17.1%) | n.a. |
| **Ceccato F et al, 2021** | 277 (157/120)  Follow-up:  biochemical and  morphological 181 patients | 122 (44%) | Cortisol level post 1mgDST >50 nmol/L | 1.9±0.7  (0.8-4.2) | Median 52 (12-175) mth | 11/181 (6.1%)  0/181 (0%) | n.a. | 0.1 (0.06-0.13) cm  ≥1 cm in 6 patients |

f, female; m, male; FU, follow-up; ACS, autonomous cortisol secretion; SD, standar deviation; IQR, interquartile range, CS, Cushing Syndrome; 1mgDST, 1-mg overnight dexamethasone suppression test; UFC, 24h urinary free cortisol levels; ACTH, adrenocorticotropic hormone levels; yrs, years; mth, months; CRH, corticotropin releasing hormone; DHEAS, dehydroepiandrosterone sulphate levels; n.a, not available; LDDST, low-dose dexamethasone suppression test; AI, adrenal incidentaloma. * Other cases: 10 patients with low DHEAS, 4 with low ACTH and 1 with high cortisol levels.
